# Bacterial Load of the Teat Apex Skin and Associated Factors at Herd Level

**DOI:** 10.3390/ani10091647

**Published:** 2020-09-14

**Authors:** Maria-Franziska Hohmann, Nicole Wente, Yanchao Zhang, Volker Krömker

**Affiliations:** 1 Microbiology, Faculty II, Hannover University of Applied Sciences and Arts, Heisterbergallee 10a, D-30453 Hannover, Germany; maria_hohmann@web.de (M.-F.H.); nicole.wente@hs-hannover.de (N.W.); yanchao.zhang@hs-hannover.de (Y.Z.); 2 Section for Production, Nutrition and Health, Department of Veterinary and Animal Sciences, Faculty of Health and Medical Sciences, University of Copenhagen, Grønnegårdsvej 2, 1870 Frederiksberg C, Denmark

**Keywords:** teat end colonization, mastitis pathogens, wet-dry swab technique, bedding, season

## Abstract

**Simple Summary:**

Bacterial load on the teat apex of dairy cows that causes intramammary infections is to a large extent due to environmental impacts. The aim of our study was to describe factors at herd level that are associated with bacterial load of environmental mastitis pathogens on the teat end’s skin. On visits to 31 dairy farms over a one-year period, farm conditions were documented, and environmental bacterial loads were examined. We found seasonal fluctuations and direct correlations between the temperature–humidity index (THI) in the barn and the bacterial load at the teat end. Significantly more environmental mastitis pathogens were found in herds with a high percentage of normal and slightly rough teat ends. The time since the last fresh bedding was added to the cubicles, as well as the frequency in which cubicles were cleaned, also affect the pathogen load on the teat skin. Pre-cleaning teats before milking as well as post-dipping after milking showed a decreasing effect of teat-skin bacterial load at the herd level.

**Abstract:**

In order to reduce antimicrobial treatment and prevent environmental mastitis, the aim of the present study was to investigate associations between herd level factors and microbial load on teat ends with environmental mastitis pathogens. Quarterly farm visits of 31 dairy farms over a one-year period were used for statistical analysis. During each farm visit, teat-skin swabs, bedding and air samples were taken and management practices and herd parameters were documented. Total mesophilic bacteria, esculin-positive streptococci and coliform bacteria were examined in the laboratory procedures from teat skin and environmental samples. Esculin-positive streptococci and coliform bacteria on teat ends increased with high temperature–humidity indices (THI) in the barn during the spring and summer. Significantly more coliform bacteria on teat ends were found in herds with an increased percentage of normal or slightly rough teat ends. Cleaning cubicles more frequently, pre-cleaning teats before milking as well as post-dipping them after milking had a decreasing effect of teat-skin load with total mesophilic and coliform bacteria at the herd level. To conclude, teat-skin bacterial load with environmental pathogens is subject to fluctuations and can be influenced by aspects of farm hygiene.

## 1. Introduction

Bovine mastitis, or inflammation of the mammary gland, is a complex disease considering its etiology and pathogenesis. As reducing antimicrobial usage is a social concern, as well as mastitis causes economic losses (reduced milk yield, discarded milk, culls, therapy costs), it is necessary to further characterize causative pathogens in order to develop control strategies [1,2]. A wide variety of microorganisms are discussed as being responsible for the development of mastitis. These can be epidemiologically categorized into contagious, originating from infected quarters or environmental, located in the surroundings of dairy cows [3,4,5,6]. While the prevalence of contagious mastitis has been reduced by control programs in recent years, environmental pathogens are becoming increasingly important [4]. Most prevalent environmental microorganisms isolated in milk samples of clinical mastitis cases occurring on German dairy farms are esculin-positive streptococci, *Escherichia coli* and *Klebsiella* spp. [7].

The teat skin seems to act as a reservoir of microorganisms, especially Gram-positive catalase-positive bacteria including coagulase-negative staphylococci [8]. Pathogenic bacteria can enter the udder through the teat canal and may cause intramammary infection (IMI). In recent years, many authors have shown that teat end bacterial load can affect udder health [9,10,11]. To gain more information concerning the variation in the bacterial load on teat epithelia, some researchers described methods quantifying teat end bacterial load. The wet-dry swab technique, described by Paduch and Krömker [12], enables a semiquantitative investigation of the teat end colonization. Some genera of physiological teat-skin flora are stated to inhibit some isolates of mastitis pathogens [13,14]. Nonetheless, generally, the microbial community of the teat surface depends on the respective farm environment. Monsallier et al. [8] showed that farming practices could interact with microbial flora on teat skin. Early on, it was recommended to reduce the environmental pathogen contamination of the teat end as a method for controlling environmental mastitis [15]. Cows spend most of the day lying down, making bedding a primary source for environmental pathogens to stick onto the teat end skin. It has been published that numeric differences in the distribution of *Streptococcus* spp., *Staphylococcus* spp., and Gram-negative bacteria on teat skin are linked to different kinds of bedding materials [6,16,17]. Furthermore, some researchers observed a reduction in teat-skin bacterial load of environmental pathogens after adding alkaline conditioner to the bedding material [18,19]. The detection of *Klebsiella* spp. from teat-skin swabs increases if udders are classified as ‘dirty’ [20]. More frequent cleaning of alley floors decrease the teat end’s coliforms and streptococci counts, as well as milk coliform counts [21,22]. Seasonal impact on teat-skin load is noted for coagulase-negative staphylococci, showing a numeric increase throughout the summer months. This effect could not be observed for the colonization of the teat canal [23]. Similar findings were reported by other authors regarding the bulk milk somatic cell count (SCC) and intramammary infection rate with environmental pathogens [24].

Factors influencing the teat-skin bacterial load appear to be well studied when considering the effect on individual level. The aim of the present study was to investigate risk factors at herd level, which are associated with higher teat-skin bacterial load of environmental pathogens in order to develop strategies to improve udder health by reducing these risk factors or adjusting farm management measures.

## 2. Materials and Methods

All applicable guidelines for the care and use of animals were followed. The study was approved by the Animal Welfare Committee of Hannover University of Applied Sciences and Arts, Germany. An application for a license for animal testing was not required by the local government. The study met the International Guiding Principles for Biomedical Research Involving Animals (1985).

### 2.1. Herds and Animals

To determine risk factors at herd level, a convenient sample of 31 conventional dairy farms in northwestern Germany were enrolled and visited from September 2018 to August 2019. Participating farms were advised by veterinarians of Hannover University of Applied Science and Arts, Germany or other veterinarians working together with the university. A total of 120 farm visits were conducted at quarterly intervals (four farms joined the trial later). Herds included mainly Holstein Friesian and Holstein crossbreeds. Farms kept 205 lactating dairy cows on average (min–max; 42–595) and had an average milk yield of 10,417 kg (7721–13,933 kg; Dairy Herd Improvement Association, DHIA) and mean SCC of 222,000 cells/mL (94,000–579,000 cells/mL; DHIA). All farms differed regarding housing, feeding and milking practices. Except for the exclusion of automatic milking systems, different conditions were noted, but were not a criterion for participation in the study.

### 2.2. Teat-Skin Swabs

Two contralateral teats (e.g., left front and right rear) were sampled with the modified wet-dry swab technique after the pre-cleaning and pre-milking routine before milking was conducted, as described by Paduch and Krömker [12]. The first swab (ultrafine, Dry Swab, Check Diagnostics GmbH, Westerau, Germany) was moistened with sterile Ringer’s solution (1/4 strength) (Merck KGaA, Darmstadt, Germany) and rotated 360° around the teat canal orifice at a distance of 1 cm from the teat apex. The same procedure was carried out with the dry swab. Immediately after sampling, the tips of both swabs were transferred to one tube containing 2 mL of sterile Ringer’s solution. Three animals (one primiparous and two multiparous cows) per farm were sampled to represent a common herd composition. All animals were in the first 30 days in milk (DIM) because this was set as the time period with the highest risk of IMI. All sampled cows were without clinical mastitis, had four functional quarters (i.e., no clotting or discoloration of milk, no swelling or udder redness and no heat upon udder palpation) and had no other illnesses, i.e., metritis or lameness. Furthermore, they had no visible udder lesions or trauma and teat skin appeared normal. Eligible cows were sampled on entering the milking parlor.

### 2.3. Bedding Samples

After milking the cows from which teat-skin swabs were taken, bedding was sampled from the pen in which these cows were housed. Wearing clean disposable gloves, used bedding was collected from four different locations per pen and then mixed in an unused glove to form a single composite bedding sample. Subsamples were taken as a grab sample from the top 8–10 cm in the back third of each cubicle. Cow pats were avoided at sampling to achieve an undistorted bedding sample. The number of days since fresh bedding had been most recently added to the pen were recorded [25].

### 2.4. Air Samples

In the same pen, an airborne dust sample was collected after cows had finished the milking session. As an active bacteria sampling technique for collecting airborne viable particles, a one-stage-impactor (AirTest OMEGA, Packhaus Rockmann GmbH, Sendenhorst, Germany) was used with an impacted volume of 10 L. The particles were impacted on an agar surface of Plate Count Agar (PC, Merck KGaA, Darmstadt, Germany), determining total mesophilic aerobic counts and Yeast Extract Glucose Chloramphenicol Agar (YGC, Merck KGaA, Darmstadt, Germany), determining yeasts and molds located below the perforated plate. The device was fixed at a height of approximately 150 cm and was located at a central place in the pen [26,27].

### 2.5. Scoring

Udder hygiene scoring (UHS) was conducted for randomly selected cows from the group of animals from which teat-skin samples had been taken and which were housed in the pen from which bedding samples had been examined. The number of cows scored varied between the farms, depending on the herd size. Scoring followed the scoring system described by Schreiner and Ruegg [28], where Score 1 was free of dirt, Score 2 was slightly dirty (2% to 10% of surface area), Score 3 was moderately covered with dirt (10% to 30% of surface area) and Score 4 was covered with caked on dirt (>30% of surface area). The percentage of cows with a clean udder (UHS 1 + 2) was included in the final statistical analysis. Teat condition scoring was conducted in accordance with the system by Mein et al. [29]; for the same cows, the UHS was determined. Teats of cows allotted to the hyperkeratosis score (HKS) 1—had no rings on the teat end, teats with HKS, 2—had a smooth or slightly rough ring, HKS 3—had a roughened ring and HKS 4—raised rings with rough fronds of old keratin. The percentage of cows classified as categories 1 and 2 were noted. Finally, the teat apex score (TAS) was determined. For this, 20 teats were pressed on a clean piece of moistened paper towel after udder preparation had been conducted by the farmer or milking personnel. The percentage of teats with dirt residues was noted, which should provide information about the effectiveness of udder preparation before milking [30].

### 2.6. Herd Management Practices

At the first farm visit, a survey was conducted. Questions were asked and observations were made about bedding management and barn hygiene (Appendix A, Table A1 and Table A2). At the following sampling events, any important management change that had occurred in-between farm visits, were noted. Latest DHIA test records from each farm visit were recorded. Additionally, morning milking was monitored during every farm visit and milking parlor routine was documented. Temperature and air humidity were recorded during the farm visit and were measured outside in front of the barn, at the feeding gate and in the cubicles, respectively. Temperature–humidity indices (THI) were calculated [31].

### 2.7. Laboratory Procedures

All samples were transported at 5 °C to the microbiology laboratory at Hannover University of Applied Sciences and Arts, Germany within 8 h. Teat-skin swabs were vortexed (Vortex Genie2, Scientific Industries, Inc., Bohemia, NY, USA) for 20 s and the liquid was pooled. Serial 1:10 dilutions were prepared with Ringer’s solution (1/4 strength) and a volume of 0.1 mL was spread in duplicate over a whole pre-dried 9-cm diameter agar plate with a Drigalski spatula in accordance with DIN 10192-5 (1995-05-00). The total number of aerobic mesophilic bacteria was determined with PC agar and incubated at 30 °C for 72 h (Table 1). Crystal-violet neutral-red bile agar (VRB, Merck KGaA, Darmstadt, Germany) was used for detecting coliform bacteria, while esculin-positive streptococci (e.g., *Streptococcus (Sc.) uberis*, *Lactococcus lactis*, *Enterococcus* spp.) were determined with Kanamycin esculin azide agar (KAA, Merck KGaA, Darmstadt, Germany). The latter two were incubated at 37 °C for 24 h. Plates with 1–300 colonies were used to calculate the weighted arithmetic mean and stated as colony-forming units per milliliter swab solution (cfu/mL) [12,19]. A total of 11 g of bedding was mixed with 99 mL autoclaved reverse osmosis water and then stomached (Fa. easy MIX, ES Laboratoire Group, Combour, France). Prepared sample material was used to determine the pH with a pH meter (DE20 FiveEasy, Mettler Toledo, Inc., Columbus, OH, USA). In the following, the sample preparations were spread out on three different culture media using the spatula method and were incubated as described above [12]. The weighted arithmetic mean was calculated and indicated in cfu/mL. Dry matter (DM) was determined by the automatic system Q-dry. Stable air agar plates were directly incubated without any dilution for 72 h at 30 °C (PC) and 25 °C (YGC), and results were reported as colony-forming units per 10 L of aspired air (cfu/10 L) (Table 1).

### 2.8. Data Management and Analysis

The collection and processing of data were carried out with Microsoft excel (Microsoft Corp., Redmond, WA, USA). For analyzing the dataset, the program SPSS 26.0 (IBM, Inc., Armonk, NY, USA) was used with herds considered as the statistical unit. To achieve statistical normal distribution, all bacterial counts were log-transformed to base 10 after adding 1 (log_10_ cfu/mL; log_10_ cfu/10 L) and tested for normal distribution with the Kolmogorov–Smirnov test. Associations between bacterial load of a pathogen group (dependent variable) and risk factors (independent variables) were examined with generalized linear mixed models after pre-screening for variable selection in univariable analysis. Dependent variables were the microbial load of teat end skin of total aerobic mesophilic bacteria, esculin-positive streptococci and total coliform bacteria as well as the ratio of esculin-positive streptococci and coliform bacteria on total mesophilic bacteria (mean of the samples in a herd at a point of time). The relation between dependent and independent variables was tested first by means of the Student’s *t*-test/Wilcoxon test/ANOVA for continuous measurements, with the exception of predictors in the same model, which indicated a correlation of r > 0.70 with one another (Spearman/Kendal’s tau; to avoid multicollinearity; for this reason, no variables were excluded). Then, independent variables associated with dependent variables at *p* < 0.10 in the univariable test were submitted to generalized linear mixed models. The subject was the herd (random) with repeated measurements. The target was the microbial load of a bacterial group in teat-skin swabs. The multivariable analysis was performed using a backward stepwise selection and elimination procedure using *p* < 0.05 for inclusion and *p* > 0.10 for exclusion. Furthermore, controlling for potential confounding variables was performed using the Wald-test *p* values. The most optimal models were evaluated using the Akaike information criterion (AIC), where an AIC closest to zero was deemed the best model. In the final models, all biologic credible two-way interactions were tested. Model fit was evaluated by checking normality of the residuals. Least square means from the models were calculated. A *p* value < 0.05 was considered indicative of a statistically significant difference.

## 3. Results

### 3.1. Description of Study Herds

Samples were collected in all four seasons (spring, summer, fall, winter) over a one-year period and participating herds represented several regions in northwestern Germany, with differing housing systems, milking parlor procedures and mastitis control practices typical of modern conventional dairy farms. The analysis included 120 records from 31 herds located in two states in northwestern Germany: Lower Saxony (n = 26) and North Rhine-Westphalia (n = 5). Herds mainly consisted of Holstein Friesian cows (mean; min–max; 94%; 20–100%).

### 3.2. Teat-Skin Swabs

On average, total mesophilic bacteria (5.00; 1.00–7.48) were most frequently isolated from the teat skin, followed by esculin-positive streptococci (2.38; 1.00–5.48), while coliforms were detected in lowest counts in the mean (1.77; 1.00–4.54). In all bacterial groups, colony counts at the lowest detection limit appeared. Coliform counts did not reach the upper detection limit as did esculin-positive streptococci. Bacterial counts of teat-skin swabs are listed in Table 2.

### 3.3. Bedding

Aerobic mesophilic counts as well as esculin-positive streptococcal and coliform counts varied in their colony counts between the detection minimum and maximum (Table 3). Bedding contained mean mesophilic bacterial counts of 9.34 log_10_ cfu/mL (1.00–11.48 log_10_ cfu/mL), mean counts of esculin-positive streptococci of 5.93 log_10_ cfu/mL (1.00–7.48 log_10_ cfu/mL) and mean coliform counts of 6.00 log_10_ cfu/mL (1.00–7.48 log_10_ cfu/mL) (Table 3). Investigation of bedding resulted in a mean pH of 8.3 (5.6–12.4) and a dry matter value of 68.8% (35.8–95.8%) for all conducted farm visits during the study period. Neither bedding bacterial load nor pH or dry matter was significantly associated with environmental teat-skin pathogen-load at herd level.

### 3.4. Airborne Dust Samples

The mean load of stable air with mesophilic bacteria was 2.01 log_10_ cfu/10 L (1.08–2.49 log_10_ cfu/10 L) and 1.49 log_10_ cfu/10 L (1.00–2.49 log_10_ cfu/10 L) for yeasts and molds (Table 4). At both agar plates, samples reached the upper detection limit. On average, total bacterial counts were more often detected than yeast and molds. Pathogen loads of air were not related to teat skin pathogen-load.

### 3.5. Scoring

The mean percentage of cows per farm with clean udders (UHS 1 + 2) and with smooth teat ends (HKS 1 + 2) was 79.8% (17.9–100%) and 88.2% (50.4–100%), respectively (Table 5). On average, 31% (0–90%) of teats still had dirt residues after preparation (TAS). With regards to HKS, when the percentage of cows scored as HKS 1 + 2 increased, the teat-skin load with coliform bacteria increased (coefficient = 0.02; *p* < 0.01). Results are shown in Table 6.

### 3.6. Farm Observations and Questionnaire

On-farm herd management practices changed during the study period and differed between farm visits (Table A1 and Table A2).

#### 3.6.1. Meteorological Data

The season when farms were visited revealed a significant impact on herds teat-skin load with absolute esculin-positive streptococci and coliform counts (*p* < 0.01) as well as with the ratio esculin-positive streptococci on total mesophilic counts (*p* < 0.01), showing an increase in the spring and summer (Table 6, Table 7 and Table 8). The THI’s at the feeding gate were positively associated with the ratio of coliform bacteria on total mesophilic counts on teat skin (coefficient = 0.01; *p* < 0.01; Table 9).

#### 3.6.2. Bedding Management

Straw accounted for 50% (n = 60) of the bedding material during the farm visits, 25.8% (n = 31) was composed of manure solids and 20.8% (n = 25) of wood shavings. Cows at remaining farm visits were housed on pasture or rubber mats all day. At 58.3% (n = 70) of visits, alkalizing conditioners were added to the bedding. New bedding was added on an average of three times/week, while 27.5% (n = 33) added bedding ≥ 3 times/week, which was more often than every second day. The mean age of used bedding samples were collected from was five milking times or 60 h, which resulted in an age of 2.5 days. In 48.7% (n = 55) of samplings, the last replacement of bedding was less than 48 h ago; at 18.6% (n = 21) of visits, the age of bedding was 49–96 h and for 17.7% of cases (n = 20), 97–144 h ago. In 15.2% (n = 17) of farm events, bedding samples had remained longer than 145 h in the cubicles. Coliform counts (*p* < 0.01) in teat-skin swabs as well as the ratio of coliform counts on total mesophilic bacteria counts (*p* < 0.05) were significantly associated with the time the bedding had been in the pen until the bedding sample was taken (= age of the bedding; Table 6 and Table 9). Lowest counts on teat skin with coliform bacteria were detected for bedding aged 97–144 h, while the lowest ratio of coliform bacteria on total mesophilic bacteria was under 48 h. No effect of the frequencies of adding new bedding was observed. With regard to cleaning the cubicles, which was done twice daily on average (0–8 times/day), the frequency of cleaning the cubicles had a decreasing effect of coliform load on teat ends at herd level (coefficient = −0.16; *p* < 0.01; Table 6).

#### 3.6.3. Milking Management

Most farms milked their cows twice daily (86.7%; n = 104 visits), while 12.5% had a milking frequency of three times/day (n = 15). During one visit, freshly calved cows were milked four times/day (0.8%), but this was subsequently discontinued. Referring to the teat’s preparation before milking, in 77.5% (n = 93) of farm visits, teats were cleaned before milking. Mostly a slightly moistened wipe was used, either one wipe/cow (31.7%; n = 38) or one wipe for more than one cow (15.8%; n = 19). During 30% of visits (n = 36), pre-dipping was conducted. The manner in which teats were prepared before milking was significantly associated with total mesophilic load in teat-skin swabs (*p* < 0.01; Table 10). Highest counts were detected in teat-skin swabs of herds where no pre-cleaning was practiced. Lower total bacterial counts were found in herds practicing pre-cleaning with one wipe/cow or with one wipe for more than one cow, although bacterial counts did not differ significantly between these methods. Pre-dipping teats, with chlorine dioxide (77.5%; n = 31), iodine (12.5%, n = 5) or lactic acid (10.0%; n = 4), resulted in lowest mesophilic counts on teats ends. After milking, in 82.5% (n = 99) of visits, a post-dip was used, which was significantly associated with a lower coliform load (*p* < 0.01) and a lower ratio of coliform bacteria on total bacteria (*p* < 0.01) than using no post-dip (Table 6 and Table 9). Most of the farms used iodine (dip or spray; 56.6%; n = 56), while 38.4% (n = 38) of farms used chlorine dioxide and 5.0% (n = 5) used lactic acid or biphenylol. There were no significant differences between these methods.

### 3.7. Statistics

Results of the final generalized linear mixed models describing risk factors associated with teat-skin load with bacteria are presented in Table 6, Table 7, Table 8, Table 9 and Table 10. Least square means describing differences between categorial risk factors are shown in Table 11.

## 4. Discussion

This is one of the first studies designed to investigate associations between herd level factors and teat-skin bacterial load. However, this was a convenient sample of herds in northwestern Germany visited from September 2018 to August 2019. The mean herd size (205 cows/farm) and milk yield (10,417 kg/cow/year) in the study populations were greater than the national average reported in the 2019 annual report of the DHIA (87 cows/farm; 8907 kg/cow/year). The mean SCC in the milk control of 222,000 SCC/mL was lower than 229,000 SCC/mL reported nationally [32]. Thus, the visited herds produced milk at a high level, which probably results from the fact that the herds consisted mainly of the dairy breed Holstein Friesian. Nevertheless, the ranges (42–595 cows/farm; 7721–13,933 kg/cow/year; 94,000–579,000 SCC/mL) may show that attempts were made to include as different herds as possible.

Swabbing surfaces to determine their bacterial load is one of the oldest methods employed for this purpose. However, our results are difficult to compare to those from existing literature because sampling methods and the culture media used for bacterial analysis were different. Some authors examined teat skins’ bacterial population by rotating or wiping one cotton or gauze swab, either dry or moistened, around the teat end [8,17,33]. Paduch and Krömker [12] modified the wet-dry swab technique (DIN 10113–1; 1997–07) used in a previous study for determining the bacterial content in milking equipment to examine the teat end’s environmental pathogen load [34]. The swab samples obtained in this way enable a semiquantitative investigation of the teat end’s colonization. In previous studies, *Staphylococcus* spp. and *Streptococcus* spp. were the predominant bacterial types recovered from teat skin, whereas Gram-negative bacteria were less numerous [17,19,35]. Our study may only partially provide this thesis as we only examined esculin-positive streptococci and coliforms. However, we also found higher mean bacterial counts of streptococci compared to coliforms. In addition, esculin-positive streptococci reached the upper detection limit, in contrast to coliforms. Therefore, it is not possible to say whether there were teat-skin samples in which higher streptococci counts appeared. Paduch et al. [19] reported similar results with the wet-dry swab technique as we did. They found that *Sc. uberis* (mean: 1.4 ± 0.2 log_10_ cfu/mL) and coliforms (mean: 1.4 ± 0.2 log_10_ cfu/mL) are always present on teat skin when housing the animals on untreated bedding, as opposed to for coliforms and enterococci (mean 0.00 log_10_ cfu/mL) in a previous study by Paduch and Krömker [12]. This may lead to the conclusion that esculin-positive streptococci belong to the normal teat-skin flora. This is supported by the results of our study showing mean streptococcal (5.93 ± 1.0 log_10_cfu/mL) and coliform (6.00 ± 1.3 log_10_ cfu/mL) counts in bedding samples to be quite similar, but revealing different counts on teat skin (2.38 ± 1.1 log_10_ cfu/mL vs. 1.77 ± 0.8 log_10_ cfu/mL), which is probably due to a shorter survival of coliforms on the teat’s surface. In the study by Paduch and Krömker [12], there were some teat-skin samples at the lowest detection limit as examined in ours, which may have resulted from, for example, bactericidal pre-cleaning, considering that teat-skin swabs were taken after a pre-cleaning routine. Differences in bacterial counts between the studies may also appear due to differing sample sizes.

Cullen and Hebert [23] recorded an increase in coagulase-negative staphylococci on teat skin in July, August and September when taking teat-skin swabs from the same cows during a trial period of some months. Since all cows included in the trial were in the same stage of lactation, it could be possible that teat-skin bacterial load increased because these animals were at the end of gestation in the summer months, produced less milk and therefore had spent more time in the stall where environmental bacteria can colonize teat skin. Cows included in our trial were in the first 30 DIM, so that we sampled different cows during every farm visit throughout the trial period. Nevertheless, there was a significant increase in mean pathogen teat-skin load with esculin-positive streptococci and coliforms in the spring and summer, which is remarkable and to our knowledge has not been previously described at herd level. THIs calculated with values measured in the barn (feeding gate) were associated with increasing ratios of coliforms on total bacteria load on teat skin. An explanation may be the impact on microbial growth by moisture, temperature as well as nutrients available on teat skin and in bedding materials. Considering the time cows spend lying down per day, pathogens may be transferred from bedding onto teat skin. In a previous study by Hughes [36], there were significantly more fecal streptococci when managing to keep the bedding surface below 15 °C and 75 % relative humidity. Both values seem more difficult to achieve in the summer months and with increasing ambient THIs. On the other hand, it could also be possible that the significantly increasing teat-skin pathogen-load in the spring and summer is less due to promoted growth than to a generally reduced time for hygiene management by the farmers since much other work must be done on a farm at this time. Individual influences by the researcher should be minimized by samples being taken by the same researcher during the study period but cannot be excluded [34]. Irrespective of the explanations for the seasonal fluctuations of esculin-positive streptococci and coliform counts on teat skin, it could be assumed that the teat and bedding management should be seasonally adjusted accordingly. Especially in summer, teats with a lowest possible bacterial load should be ensured, as cows are much more susceptible to environmental mastitis at this time [24]. Furthermore, the aim should be to achieve an as low as possible THI in the barn (e.g., ventilation/cooling) in order to reduce environmental pathogen load on the teat dip, but also to minimize other effects associated with heat stress in dairies [37].

Even if our results show no significant impact, previous studies required management strategies to obtain an as low as possible bacterial load in bedding material to reduce teat-skin bacterial load and prevent environmental mastitis [19,38,39]. Therefore, Krömker et al. [40] published benchmarks for bacterial counts in unused bedding, indicating a reduced risk for cows to develop mastitis: For esculin-positive streptococci and coliforms: 10^4^ cfu/g and for total bacteria counts: 10^6^ cfu/g (sawdust) or 7 × 10^8^ cfu/g (straw). In a recently published study, achievable benchmarks for used bedding were published: For streptococci-like organisms (SSLO): 5 × 10^5^ cfu/cm^3^ and for coliforms: 10^4^ cfu/cm^3^ [25]. Data from previous studies dealing with bacterial growth in bedding materials indicated a nonlinear relationship between time and bacterial counts but a maximum increase within 24 to 36 h of use following contamination of animals and feces [38,41]. Fewer changes in bacterial counts in bedding material were recorded after 24 h. Similar results were found by Hogan and Smith [42] Streptococcal counts, *Klebsiella* spp. counts, pH and DM in bedding did not differ between days 1–6, while coliforms were greater at day 1 than at day 6. These steady or decreasing bacteria counts implied that bacteria decreased their growth cycle. In a previous study, it was reported that bacteria counts tended to be lower after extended use than bedding counts within the first days after adding it to the cubicles [43]. As can be seen from our results, considering the age of bedding, total coliforms were more or less unchanged the older the bedding was. On the other hand, the ratio of coliforms on total bacteria counts was lowest under 48 h, thereafter, showing the most significant increase, which may indicate a decrease in the total bacteria—even if this cannot be shown for absolute total bacteria counts. It may be necessary to narrow down the number of categories in order to detect changes before 48 h. However, in our trial, esculin-positive streptococci on teat skin were not influenced by the age of bedding, therefore drawing the conclusion that streptococci are not influenced by the environment as much as previously thought. Other interactions must be considered, as different types of bedding may enhance the growth of different pathogens [44,45] or adding lime to the bedding reduces the population sizes of environmental pathogens [19]. Furthermore, the cleaning of passageways can reduce muck and slurry being transferred to cows’ cubicles and teats via the cows’ feet [46]. Statistical analysis revealed no significant associations when including these interactions. From our results can be seen that bedding profoundly affects the microbiological population on teat skin. As supported by our results, Sorter et al. [39] showed that daily bedding replacement in the rear of the cubicles decreased teat exposure to coliform bacteria, but not to streptococci. From these findings, it can be deduced that daily bedding replacement is necessary to keep the bacterial counts on the teat skin and bedding as low as possible or, if this cannot be implemented, to use bedding with the lowest possible initial bacterial load in unused bedding. Considering the seasonally increased pathogen load on teat skin, it is advisable, especially in spring and summer months to achieve the lowest possible microbial load in bedding materials. Thus, for example, adding lime, using inorganic bedding materials and daily bedding replacement in the rear of the cubicles appears to be most advantageous during this time. In contrast to the replacement interval for bedding, the interval in which the lying area was cleaned had a direct effect on the environmental pathogen load on cows’ teat ends. Coliform bacteria on teat skin decreased the more the cubicles were cleaned per day. Similar results were published by a British study, demonstrating that when collected yards were cleaned at least twice daily a small protective effect on the mastitis incidence could be determined [47]. This indicates that it seems to be as important to keep the bedding clean as it is to constantly replace it in order to remove organic nutrients that promote pathogenic growth.

There are few published results on total aerobic mesophilic count, which is not surprising as it contains both pathogenic and nonpathogenic bacteria. The latter are not very informative, when considering the reduction in IMIs. This can be confirmed by our results, showing significant associations among herd risk factors and teat end environmental pathogen load, e.g., season, age of bedding or post-dip, which cannot be seen for total mesophilic counts. Therefore, it seems irrelevant when investigating the influence of total bacterial load on teat skin regarding the risk of IMI. The generalized linear mixed model described a significant influence of teats preparation before milking on total bacterial count on teat skin. Not surprisingly, considering that the teat skin was sampled directly after pre-milking and pre-cleaning, highest values occurred on farm visits where no pre-cleaning of teats was conducted. However, examining the influence of pre-cleaning on mesophilic bacteria load could be useful when describing the efficiency of teat cleaning [17]. Pre-milking teat disinfection is practiced in several countries to reduce the microbial load of the teats prior to milking and to prevent mastitis caused by environmental pathogens. We observed that usage of disinfectant pre-dip is associated with significantly lower counts of total mesophilic bacteria than when practicing no pre-cleaning routine, which is associated with highest bacterial counts on teat skin. Pre-dip is a demonstrated and widely accepted practice to reduce teat-skin environmental-load and control environmental mastitis [48]. We expected lower bacterial loads when using one wipe for one cow as when using one towel for more than one cow, hypothesizing that dirt residues were transferred from teat-to-teat. However, no significant differences could be seen. We assume that this observation is due to our small sample size, considering that the impact of one farm in our statistics was quite high. Therefore, such results can be obtained from farms that generally have higher levels of pathogens in their environment that try to correct these pathogen levels by implementing a more accurate teat-preparation before milking. Results may be different if the trial had been conducted only on farms with a higher hygiene level. In a previous study, pre-milking routine was not able to remove *Klebsiella* spp. from teat skin, especially when udders were dirty. Even when udder preparation procedures include the use of teat disinfectants, they may not be effective in disinfecting the teats of cows with udders that would be classified as category 3 or above [20]. Nevertheless, teats should be pre-cleaned before milking to achieve lower pathogen loads on teat skin, as all procedures were associated with lower teat end bacterial load than not pre-cleaning the teats.

Usage of teat dips after milking reduced coliforms and the ratio of coliforms in total aerobic mesophilic counts. Considering that teat dipping had been conducted after milking time, before teat-skin samples were taken, using a post-dip seems to have a long-lasting effect on teat-skin pathogen-load. Interestingly, this bactericidal effect does not occur in absolute esculin-positive streptococci counts. Some authors indicated no long-lasting effect of post-dips against *Sc. uberis* than *Staphylococcus (S.) aureus,* explaining this by saying that *S. aureus* is not widely distributed in the environment so that once removed by disinfection it rarely leads to a recontamination [49]. This does not explain our results, as coliforms as well as esculin-positive streptococci were frequently found in the dairy’s surroundings. Perhaps it can be again suggested that streptococci are a part of facultative teat-skin flora, so that no significant effect of bactericidal treatment after milking can be recognized at the following milking session. Other farm factors may also play a role here, so that implementing a post-dip is an indication of a generally higher standard of on-farm hygiene. However, the usage of a post-dip after milking is strongly recommended based on our results.

In previous studies, results regarding the influence of hyperkeratosis and udder health differ. The study by Paduch et al. [50] indicated that *E. coli* counts in teat canal swabs are significantly associated with the teat ends hyperkeratosis score. Some authors associated higher levels of hyperkeratosis with increasing numbers of intramammary infections, as rough teat apex surfaces are more difficult to clean and are often associated with teat end lesions, leading to a more frequent colonization with bacteria [11,51]. However, Guarin et al. [10] could not find any association between hyperkeratosis and teat-skin load with environmental pathogens. Zoche-Golob et al. [52] could not observe any variable describing teat condition on the risk of developing mastitis. According to our results, coliform bacterial load on teat ends increases as the percentage of cows with normal and healthy teat apices among all lactating cows per herd increases. We could not find any other previous study that has found this correlation. Our results can be explained with findings by Neijenhuis et al. [51], who reported that cows with clinical mastitis caused by other pathogens other than *E. coli* (e.g., *S. aureus,* coagulase-negative staphylococci) exhibited more teat end callosity. In another study, *S. uberis* was most frequently isolated from foremilk samples from cows with HKS 1, while coagulase-negative staphylococci were most frequently isolated from cows with HKS 4 [53]. With regards to our results, it seems probable that rough teat ends are colonized by microorganisms other than those we studied, for example, *Staphylococcus* spp. Subsequently, coliform bacteria were relatively more frequent isolated from herds with a higher percentage of normal teat ends. However, the HKS was not determined in all cows in a herd. In fact, udders were scored on cows that were milked, while teat-skin swabs were taken from cows within the first 30 DIM. Accordingly, either a convenient sample of the herd or an extra group of freshly calved cows was scored, depending on herd size and herd management. This resulted in a preselection and relatively more freshly calved cows that were scored, which may have had lower hyperkeratosis scores and higher teat-skin pathogen-loads, possibly while being housed in a separated resting area. Therefore, our study design may have led to this finding, as the HKS should be determined in all cows in the herd.

It can be concluded that teat-skin bacterial load with environmental mastitis pathogens is to a large extent due to environmental impact, whereby we mainly concentrated on the hygienic aspects at herd level in the present study—namely, teat sanitation, bedding hygiene and air dust pathogen load. In order to identify further risk factors for increased exposure of the teat skin to pathogens, considering management in more detail, further research with a more targeted study design is required. Moreover, research is needed to prove the impact of teat-skin bacterial load on intramammary infection rate at herd level.

## 5. Conclusions

Taking into account that a convenient sample of farms was included in this herd level study, it can be seen that teat-skin bacterial load with environmental pathogens is subjected to fluctuations and can be influenced by farm hygiene. The seasonally fluctuating load on the teat skin from coliforms and environmental streptococci, which is higher in spring and summer, requires seasonally adapted teat management. Pre-cleaning teats before milking and dipping teats after milking can reduce bacterial load in the short- and long-term. Bedding management influences pathogenic bacterial load on teat skin significantly so daily replacement and frequent cleaning of the lying area are needed to decrease bacteria on teat skin.

## Figures and Tables

**Table 1:** Overview of the laboratory analyses carried out.

Parameter	Agar	Incubation	Unit
Wet-dry swabs			
Aerobic mesophilic bacteria	PC ^1^	30 °C, 72 h	cfu/mL ^4^
Esculin-positive streptococci	KAA ^3^	37 °C, 24 h	cfu/mL
Coliform bacteria	VRB ^2^	37 °C, 24 h	cfu/mL
Bedding samples			
Aerobic mesophilic bacteria	PC	30 °C, 72 h	cfu/mL
Esculin-positive streptococci	KAA	37 °C, 24 h	cfu/mL
Coliform bacteria	VRB	37 °C, 24 h	cfu/mL
Airborne dust samples			
Aerobic mesophilic counts	PC	30 °C, 72 h	cfu/10 L ^5^
Yeasts and molds	YGC	25 °C, 72 h	cfu/10 L

^1^ plate count agar. ^2^ crystal-violet neutral-red bile agar. ^3^ kanamycin esculin azide agar. ^4^ colony-forming units per milliliter swab or bedding solution. ^5^ colony-forming units per 10 L of aspired air.

**Table 2:** Bacterial counts (log_10_ cfu/mL) of 120 pooled wet–dry teat-skin samples.

Parameter	Mean	Minimum	Maximum
Aerobic mesophilic counts	5.00	1.00	7.48
Esculin-positive streptococci	2.38	1.00	5.48
Coliform bacteria	1.77	1.00	4.54

**Table 3:** Bacterial counts (log_10_ cfu/mL), pH and dry matter (%) of 120 bedding samples.

Parameter	Mean	Minimum	Maximum
Aerobic mesophilic bacteria	9.34	1.00	11.48
Esculin-positive streptococci	5.93	1.00	7.48
Coliform bacteria	6.00	1.00	7.48
pH	8.3	5.6	12.4
DM ^1^	68.8	35.8	95.8

^1^ Dry matter.

**Table 4:** Bacterial counts (log_10_ cfu/10 L) of 120 airborne dust samples.

Parameter	Mean	Minimum	Maximum
Aerobic mesophilic counts	2.01	1.08	2.49
Yeasts and molds	1.49	1.0	2.49

**Table 5:** Results (%) of 120 herd udder scorings.

Independent Variable	Mean	Minimum	Maximum
UHS 1 + 2 ^1,2^	79.8	17.9	100
HKS 1 + 2 ^3,4^	88.2	50.4	100
TAS ^5,6^	31	0	90

^1^ udder hygiene score—UHS 1—free of dirt, UHS 2—slightly dirty, UHS 3—moderately covered with dirt, UHS 4—covered with caked on dirt [28]. ^2^ percentage of cows with UHS 1 + 2 in all examined cows. ^3^ hyperkeratosis score—HKS 1—no ring on teat end; HKS 2—smooth or slightly rough ring; HKS 3—roughened ring; HKS 4—raised rings with rough fronds of old keratin [29]; ^4^ Percentage of cows with HKS 1 + 2 in all examined cows; ^5^ teat apex score; ^6^ percentage of teats with dirt residues after udder preparation before milking [30].

**Table 6:** Final generalized linear mixed model based on 120 farm visits describing risk factors (= independent variable) associated with log_10_-transformed teat-skin load at herd level with coliform bacteria (= dependent variable).

Independent Variable	Data Form	Categories	Coefficient	SE ^1^	*p* Value	95% CI ^2^
Season	Categorical	Spring	0.066	0.165	0.690	−0.262–0.394
		Summer	0.413	0.205	0.047	0.005–0.820
		Fall	−0.216	0.173	0.215	−0.560–0.128
		Winter	0 *			
Frequency of cleaning cubicles	Discrete		−0.164	0.052	0.002	−0.267–−0.060
Age of bedding ^3^	Categorical	1–48 h	−0.216	0.177	0.226	−0.568–0.136
		49–96 h	−0.291	0.214	0.178	−0.72–0.14
		97–144 h	−0.700	0.200	0.001	−1.096–−0.303
		>145 h	0 *			
Percentage of HKS 1 + 2 ^4^ per herd	Discrete		0.015	0.006	0.008	0.004–0.026
Post-dip	Categorical	None	1.353	0.768	0.081	−0.172–2.877
		Iodine (spray)	0.882	0.766	0.252	−0.639–2.403
		Iodine (dip)	0.860	0.755	0.258	−0.639–2.358
		Chlorine dioxide	0.536	0.755	0.480	−0.965–2.036
		Lactic acid	0.021	0.808	0.980	−1.585–1.626
		Biphenylol	0 *			

^1^ standard error; ^2^ confidence interval; ^3^ time between last replacement and sampling of bedding; ^4^ hyperkeratosis score: HKS 1—no ring on teat end; HKS 2—smooth or slightly rough ring; HKS 3—roughened ring; HKS 4—raised rings with rough fronds of old keratin [29]; * redundant coefficient set to zero.

**Table 7:** Final generalized linear mixed model based on 120 farm visits describing risk factors (= independent variable) associated with log_10_-transformed teat-skin load at herd level with esculin-positive streptococci (= dependent variable).

Independent Variable	Data Form	Categories	Coefficient	SE ^1^	*p* Value	95% CI ^2^
Season	Categorical	Spring	0.478	0.184	0.010	0.114–0.842
		Summer	1.111	0.252	0.000	0.613–1.610
		Fall	0.071	0.177	0.687	−0.279–0.422
		Winter	0 *			

^1^ standard error. ^2^ confidence interval. * redundant coefficient set to zero.

**Table 8:** Final generalized linear mixed model based on 120 farm visits describing risk factors (= independent variable) associated with the ratio of esculin-positive streptococci on total mesophilic teat-skin load on herd (= dependent variable).

Independent Variable	Data Form	Categories	Coefficient	SE ^1^	*p*-Value	95% CI ^2^
Season	Categorical	Spring	0.140	0.038	0.000	0.064–0.215
		Summer	0.234	0.042	0.000	0.150–0.318
		Fall	0.078	0.050	0.120	−0.021–0.178
		Winter	0 *			

^1^ standard error. ^2^ confidence interval. * redundant coefficient set to zero.

**Table 9:** Final generalized linear mixed model based on 120 farm visits describing risk factors (= independent variable) associated with the ratio of coliform bacteria on total aerobic mesophilic teat-skin load on herd level (= dependent variable).

Independent Variable	Data Form	Categories	Coefficient	SE ^1^	*p* Value	95% CI ^2^
THI ^3^ at the feeding gate	Continuous		0.005	0.002	0.002	0.002–0.009
Age of bedding ^4^	Categorical	1–48 h	−0.086	0.039	0.030	−0.163–−0.009
		49–96 h	0.015	0.049	0.763	−0.082–0.112
		97–144 h	−0.032	0.050	0.513	−0.131–0.066
		>145 h	0 *			
Post-dip (categorized)	Categorical	None	0.104	0.036	0.004	0.033–0.175
		Yes	0 *			

^1^ standard error. ^2^ confidence interval. ^3^ temperature–humidity-index = (1.8 × T °C + 32) − (0.55 − 0.0055 × RH%) × (1.8 × T °C − 26) [31]. ^4^ time between last replacement and sampling of bedding. * redundant coefficient set to zero.

**Table 10:** Final generalized linear mixed model based on 120 farm visits describing risk factors (= independent variable) associated with log_10_-transformed teat-skin load at herd level with total aerobic mesophilic bacteria (= dependent variable).

Independent Variable	Data Form	Categories	Coefficient	SE ^1^	*p* Value	95% CI ^2^
Pre-cleaning of teats	Categorical	None	1.159	0.374	0.002	0.419–1.899
		One wipe/>one cow	0.707	0.372	0.060	−0.030–1.444
		One wipe/cows	1.155	0.320	0.000	0.522–1.788
		Pre-dip	0 *			

^1^ standard error. ^2^ confidence interval. * redundant coefficient set to zero.

**Table 11:** Least square means describing differences between categorical risk factors (= independent variable) associated with environmental pathogen teat-skin load at herd level (= dependent variable).

Dependent Variable	Independent Variable	Categories		Mean	SE ^1^	95% CI ^2^
Total aerobic mesophilic bacteria	Pre-cleaning of teats	None		5.442	0.292	4.864–6.020
		One wipe/>one cow		4.990	0.312	4.372–5.608
		One wipe/cows		5.438	0.239	4.964–5.912
		Pre-dip		4.283	0.233	3.821–4.746
Esculin-positive streptococci	Season	Spring		2.420	0.166	2.091–2.749
		Summer		3.053	0.239	2.579–3.527
		Fall		2.013	0.159	1.698–2.329
		Winter		1.942	0.176	1.593–2.291
Coliform bacteria	Season	Spring		1.509	0.167	1.177–1.842
		Summer		1.856	0.207	1.444–2.267
		Fall		1.227	0.176	0.878–1.576
		Winter		1.443	0.180	1.086–1.800
	Age of bedding ^3^	1–48 h		1.594	0.151	1.295–1.894
		49–96 h		1.519	0.207	1.1.08–1.930
		97–144 h		1.111	0.196	0.721–1.500
		>145 h		1.810	0.197	1.420–2.200
	Post-dip	None		2.253	0.145	1.965–2.541
		Iodine (spray)		1.782	0.147	1.491–2.074
		Iodine (dip)		1.760	0.127	1.507–2.012
		Chlorine dioxide		1.436	0.121	1.196–1.676
		Lactic acid		0.921	0.324	0.277–1.565
		Biphenylol		0.900	0.752	−0.593–2.393
Ratio of esculin-positive streptococci on total mesophilic bacteria	Season	Spring		0.511	0.036	0.439–0.583
		Summer		0.606	0.041	0.525–0.686
		Fall		0.450	0.049	0.353–0.547
		Winter		0.372	0.031	0.310–0.433
Ratio of coliform bacteria on total mesophilic bacteria	Age of bedding ^3^	1–48 h		0.343	0.023	0.298–0.389
		49–96 h		0.444	0.036	0.372–0.516
		97–144 h		0.397	0.036	0.326–0.467
		>145 h		0.429	0.036	0.357–0.501
	Post-dip (categorized)	None		0.455	0.032	0.392–0.519
		Yes		0.351	0.017	0.317–0.386

^1^ standard error. ^2^ confidence interval. ^3^ time between last replacement and sampling of bedding.

## Appendix A

**Table A1:** Farm-level associated risk factors and their mean, minimum and maximum values considered in the univariate analysis of their associations with teat-skin bacterial loads of aerobic mesophilic counts, esculin-positive streptococci and coliform counts.

Risk Factor	Minimum	Maximum	Mean
Herd traits ^1^			
Cows at ketosis risk (%) ^2^	0.0	64.1	64.1
Cows at acidosis risk (%) ^3^	0.0	56.8	56.8
Management traits			
Replacement of bedding (x/week)	1	21	3
Cleaning cubicle (x/day)	0	8	2
Cleaning walkways (x/day)	0	24	9.2
Cleaning alleyways (x/day)	0	24	2.8
Trough surface (cm/cow)	1.1	12.8	6.7
Cleaning troughs (x/week)	0	7	3.2
Meteorological data			
Temperature outside (°C)	−7	24.5	8.4
Temperature at feeding gate (°C)	−2.5	23.8	9.4
Temperature in cubicle (°C)	1.6	24.5	9.8
Relative humidity (RH) outside (%)	40	88	66.2
RH at feeding gate (%)	41	89	68.1
RH in cubicle (%)	42	92	69.3
Temperature–humidity-index (THI) ^4,^ outside	30.4	73.4	49.4
THI at feeding gate	34.9	71.9	50.7
THI in cubicle	36.1	72.9	51.3

IMIR—intramammary infection rate; SCC—somatic cell count; ^1^ latest Dairy Herd Improvement Association test records at every farm visit; ^2^ fat–protein ratio in milk > 1.5 in cows within first 100 DIM/all cows within first 100 DIM [54]; ^3^ fat-protein-ratio in milk < 1.1 in lactating cows/all lactating cows [54]; ^4^ temperature–humidity-Index = (1.8 × T °C + 32) − (0.55 − 0.0055 × RH%) × (1.8 × T °C − 26) [31].

**Table A2:** Farm-level-associated risk factors and their percentage of all farm visits considered in the univariate analysis of their associations with teat-skin bacterial loads of aerobic mesophilic counts, esculin-positive streptococci and coliform counts.

Risk Factor	Total Samples (no.)	Percentage of All Samples (%)
Lactating housing type		
Deep-bedded cubicles	101	84.2
Raised bedded cubicles	15	12.5
Deep litter	4	3.3
Percentage of dirty cubicles/all cubicles		
<10%	59	49.2
≥10%	35	29.2
≥20%	11	9.2
≥30%	1	0.8
Bedding material		
Straw	60	50
Manure solids	31	25.8
Wood shavings	25	20.8
Usage of alkalizing additives		
Yes	70	58.3
No	48	40
Days cows spend in separated pens after calving		
0 d	20	16.7
1–3 d	25	20.8
4–7 d	22	18.3
8–14 d	22	18.3
15–21 d	15	12.5
>21 d	16	13.3
Storage of bedding		
Farm buildings	53	44.2
Covered by fleece/foil	35	29.2
Fresh manure solids	27	22.5
Uncovered	5	4.1
Age of bedding samples ^1^		
1–48 h	55	48.7
49–96 h	21	18.6
97–155 h	20	17.7
>145 h	17	15.2
Order of milking fresh calved cows		
Among all lactating cows	76	63.3
At the beginning	44	36.7
Pre-cleaning teats		
One cow/wipe	38	31.7
Pre-dip	36	30.0
None	27	22.5
More cows/wipe	19	15.8
Pre-dip		
Chlorine dioxide	31	77.5
Iodine (dip)	5	12.5
Lactic acid	4	10.0
Pre-milking		
On the floor	98	81.6
None	14	11.6
Pre-cup	8	6.7
Disinfection of milking equipment		
None	88	73.3
In-between each cow	23	19.2
In-between different groups	9	7.5
Using post-dips		
Yes	99	82.5
No	21	17.5
Post-dip		
Iodine (dip or spray)	56	56.6
Chlorine dioxide	38	38.4
Lactic acid	4	4.0
Biphenylol	1	1.0
Fly control		
None	107	89.1
Cow treatment	7	5.9
Environmental treatment	6	5.0

^1^ time between last replacement and sampling of bedding material.
